# Time domain measures of inter-channel EEG correlations: a comparison of linear, nonparametric and nonlinear measures

**DOI:** 10.1007/s11571-013-9267-8

**Published:** 2013-09-04

**Authors:** J. D. Bonita, L. C. C. Ambolode, B. M. Rosenberg, C. J. Cellucci, T. A. A. Watanabe, P. E. Rapp, A. M. Albano

**Affiliations:** 1 Department of Physics, Mindanao State University-Iligan Institute of Technology, 9200 Iligan City, Philippines; 2 Thomas Jefferson University College of Medicine, Philadelphia, PA USA; 3 Aquinas, LLC, Berwyn, PA 19312 USA; 4 Lannister-Finn, Bryn Mawr, PA 19010 USA; 5 Department of Military and Emergency Medicine, Uniformed Services University of the Health Sciences, Bethesda, MD 20814 USA; 6 Physics Department, Bryn Mawr College, Bryn Mawr, PA 19010 USA

**Keywords:** EEG, Quantitative EEG, Pearson product moment correlation, Spearman rank order correlation, Kendall rank order correlation, Mutual information

## Abstract

Correlations between ten-channel EEGs obtained from thirteen healthy adult participants were investigated. Signals were obtained in two behavioral states: eyes open no task and eyes closed no task. Four time domain measures were compared: Pearson product moment correlation, Spearman rank order correlation, Kendall rank order correlation and mutual information. The psychophysiological utility of each measure was assessed by determining its ability to discriminate between conditions. The sensitivity to epoch length was assessed by repeating calculations with 1, 2, 3, …, 8 s epochs. The robustness to noise was assessed by performing calculations with noise corrupted versions of the original signals (SNRs of 0, 5 and 10 dB). Three results were obtained in these calculations. First, mutual information effectively discriminated between states with less data. Pearson, Spearman and Kendall failed to discriminate between states with a 1 s epoch, while a statistically significant separation was obtained with mutual information. Second, at all epoch durations tested, the measure of between-state discrimination was greater for mutual information. Third, discrimination based on mutual information was more robust to noise. The limitations of this study are discussed. Further comparisons should be made with frequency domain measures, with measures constructed with embedded data and with the maximal information coefficient.

## Introduction

The connectivity of the human central nervous system is its most distinctive feature. Classically connectivity was investigated anatomically. An alternative view emerged in the twentieth Century which emphasized the movement of information. Like many concepts, the seemingly straightforward idea of connectivity was found to be far more complicated than originally anticipated when it was examined with sufficient care. This can be seen in the report of the 2002 Functional Connectivity Workshop (Lee et al. 2003). Three distinct conceptualizations of connectivity have emerged: anatomical, functional and effective. Anatomical complexity might seem to be the least problematical, and arguably it is, but nonetheless complications present themselves. A complete anatomical description requires not merely knowledge of geometrical proximity but an understanding of receptor subtypes and the availability of neurotransmitters (Lee et al. 2003). Functional connectivity is defined as the “temporal correlations between spatially remote neurophysiological events” (Friston et al. 1993a), and effective complexity is defined as “the influences that one neural system exerts over another either directly or indirectly” (Friston et al. 1993b). Horowitz (2003), using the word “elusive,” found that all three conceptualizations of connectivity present subtleties of definition and that these problems were compounded when an attempt was made to integrate results obtained from different observational technologies. His analysis led to three conclusions. First, “we should think of functional (and effective) connectivity not as a single concept or quantity, but rather as forming a class of concepts with multiple members.” Second, “functional and effective connectivity must be operationally defined by each investigator who evaluates these quantities.” Third, “it is crucial to relate each of the macroscopic definitions to an underlying neural substrate.”

Fingelkurts et al. (2005) concurred in recognizing that theoretical and methodological clarifications are needed to bring precision to the analysis of CNS connectivity. They argue that the time scale of neuroanatomical change is such that an examination of anatomical connectivity cannot provide a basis for a dynamical investigation of perceptual and cognitive processes. They further argue that effective connectivity is identified by first establishing functional connectivity and combining it with a model specifying the causal links between participating units. They therefore conclude that “functional connectivity is the most central and challenging of the three conceptions of brain connectivity for theories about neural interactions.” Given the millisecond time scale of dynamical behavior in the central nervous system, Fingelkurts et al. argue for an essential role of EEG and MEG in investigations of functional connectivity. We concur, and the analysis of temporal correlations of EEG signals is the focus of this contribution. Four time domain procedures for quantifying correlations are compared. A physiological criterion, the ability to discriminate between behavioral states, is used as an adjudicating criterion. Additional measures that should be incorporated in an expanded study are considered in the “Discussion” section of this paper.

When using scalp EEG signals in the analysis of functional connectivity an additional question should be considered. Can the analysis be conducted with the original scalp signals, or is it essential to transform these signals to provide an estimate of the current source density? It is not our present purpose to participate in this debate. Conclusions about the comparative effectiveness of different measures for identifying correlations in scalp signals, which is our objective, will be applicable to calculations with current source density estimates. Two additional observations in this regard can be made. First, in practice, calculations should be performed with both original voltage signals and with transformed signals, and the results should be compared. Second, we should bear in mind Horwitz’s very valuable observation that each investigator should define the operational definition of connectivity being implemented.

The earliest example of interregional EEG correlation measurement that has come to our attention is Imahori and Suhara (1949 cited by Gevins 1987) where hand calculated autocorrelations of short EEG segments were presented. The use of autocorrelation and cross-correlations to study electroencephalograms is reported to have been suggested by Norbert Wiener in 1949 to a group of researchers at the Massachusetts General Hospital (Barlow 1997). Among this group were Mary Brazier and James Casby who in 1950 started their pioneering work on correlation analysis of the EEG using an electronic digital correlator at the Massachusetts Institute of Technology (Brazier and Casby 1952). An important continuing application of cross-correlation calculations is the correlation of EEGs with templates of averaged event related potentials where the procedure is used to locate single trial event related potentials, ERPs, in background EEG signals (McGillem and Aunou 1987 reviewed by Spencer 2005). This procedure was introduced by Woody (1967) to detect epileptic spikes. It was first applied to ERP signals by Kutas et al. (1977). This method continues to be applied in the analysis of epileptic seizures (Filligoi et al. 2011) and in the construction of brain computer interfaces (Cabestang et al. 2007).

The study of CNS correlations evolved to include more sophisticated measures. An important step in this evolutionary process was the introduction of mutual information, a nonlinear measure of correlation, to the analysis of EEGs. The earliest application of mutual information in electroencephalography that we have seen is Callaway and Harris (1974) where it was called the coefficient of information transmission. In this application, mutual information was not calculated directly from voltage time series. Digitizing at 250 Hz, each entry was coded for polarity (positive or negative) and derivative (increasing or decreasing). Callaway and Harris showed that a reading task increased occipital to left hemisphere coupling while a visual processing task increased occipital to right hemisphere coupling. In a subsequent publication (Yagi et al. 1976), Callaway and his colleagues investigated the sensitivity of this measure to epoch length and sampling frequency. Mars and Lopes da Silva (1987) showed that mutual information can identify significant correlations that are not detected by linear measures. Other applications of this measure in electroencephalography were published by Xu et al. (1997), Albano et al. (2000) and Chen et al. (2000). A limiting factor in use of mutual information has been data requirements for the estimation, computational times and uncertainty about the accuracy of the estimate. This point is addressed presently.

While being a problem of general interest in CNS physiology, the quantitative characterization of interregional correlations are of particular importance in the study of traumatic brain injury. The development of current thought about functional connectivity following TBI has many contributors, but two individuals who must appear in any account of this historical process are John Hughlings-Jackson (1835–1911) and Kurt Goldstein (1878–1965). Hughlings-Jackson and Goldstein both concluded that the recovery of function, typically partial recovery, following brain injury argued against a strong localization model of CNS organization (Hughlings-Jackson 1874, 1882; Goldstein 1934). In addition to rejecting strong localization, Goldstein’s work with CNS injured soldiers following World War I led him to conclude that recovery did not result from repair but rather from adaptation (Zeitlinger 2001). Hughlings-Jackson’s and Goldstein views concerning nonlocalization of deficit are consistent with recent research identifying failures of distributed synchronous networks in the etiology of neuropsychiatric disorders (Herrmann and Demiralp 2005; Schnitzler and Gross 2005; Stam 2005; Uhlhaas and Singer 2006). While Goldstein’s views on the failure of repair and his emphasis on adaptation following traumatic brain injury must be reconsidered in the light of the discovery of neurogenesis in the adult mammal, evidence indicates that at least for the immediate present they are still essentially correct. This process of adaptation would, one predicts, result in altered patterns of correlations in the post-injury central nervous system. This expectation has been realized in the recent literature (see Table 1 below, these are representative examples drawn from a large literature). In summary, studies of altered functional connectivity following traumatic brain injury utilize three kinds of data, EEG signals, MEG signals and fractional anisotropy measures of axonal tracts characterized by diffusion tensor imaging. This contribution is directed to EEG-based assessments. Three classes of analysis measures are used in these EEG studies, time domain measures, frequency domain measures and measures constructed with embedded data. The focus here is on time domain measures. We explicitly recognize that further comparative studies should include the additional measures described in the “Discussion” section of this paper.

**Table 1:** Pathological conditions associated with altered functional connectivity (representative examples)

Alzheimer’s disease	Georgopoulos et al. (2007), Güntekin et al. (2008), Locatelli et al. (1998), Rosenbaum et al. (2008), Stam et al. (2006, 2007a, b 2009), Zhou et al. (2008)
Epileptic seizures	Ponten et al. (2007)
Intra-arterial amobarbital injection	Douw et al. (2010)
Autism spectrum disorder	Belmonte et al. (2004), Just et al. (2004), Kana et al. (2007), Murias et al. (2007), Rippon et al. (2006), Vidal et al. (2006)
Brain tumors	Bartolomei et al. (2006), Bosma et al. (2008)
Multiple sclerosis	Georgopoulos et al. (2007), Lenne et al. (2012)
Preterm birth	Mullen et al. (2011)
PTSD	Lanius et al. (2004), Shaw 2002
Schizophrenia	Breakspear et al. (2003), Georgopoulos et al. (2007), Lawrie et al. (2002), Lynall et al. (2010), Michelyannis et al. (2006), Symond et al. (2005)
Stroke	Grefkes and Fink (2012)
Traumatic brain injury	Cao and Slobounov 2010), Castellanos et al. (2010, 2011a, b), Ham and Sharp 2012), Kasahara et al. (2010), Kumar et al. (2009), Nakamura et al. (2009), Sponheim et al. (2011), Tsirka et al. (2011)

## Correlation measures assessed

Four time domain measures for quantifying relationships between time series are compared in this investigation: Pearson product moment correlation, Spearman rank order correlation, Kendall rank order correlation and mutual information. These measures will be used to quantify between-channel correlations in EEGs recorded from healthy participants in two behavioral conditions: eyes open, no task and eyes closed, no task. The psychophysiological utility of each measure is assessed by determining its ability to discriminate between these conditions.

A brief presentation of the mathematical properties of these measures is given in the “Appendix”. Qualitative descriptions are given here. The Pearson product moment correlation quantifies linear correlations between variables. The Spearman rank order correlation is the product moment correlation of ranks, and the Kendall rank order correlation uses the relative ordering of ranks. The mutual information of two time series is the average number of bits of each that can be predicted by measuring the other. The numerical estimation of mutual information can be computationally demanding, and the accuracy of the estimate can be sensitive to the algorithm used. This was demonstrated by the comparison studies conducted by Quian Quiroga et al. (2002) and by Duckrow and Albano (2003). In a valuable study, Quian Quiroga et al. compared five measures of interhemispheric correlations (nonlinear dependencies, phase synchronization, mutual information, cross correlation and coherence). Except for mutual information, the measures showed qualitatively similar results, and, importantly the computations identified interhemispheric dependencies that were not apparent on conventional visual examination performed by a Board certified electroencephalographer. Quian Quiroga et al. used a fixed bin-width histogram method for estimating the joint probability distributions. Estimating the joint probability distribution is a critical element in the estimation of mutual information (see the “Appendix” for the mathematical details). Using the same data, Duckrow and Albano used the Fraser–Swinney (1986) adaptive partition when estimating joint probability distributions. This computation of mutual information produced results consistent with the other measures. Several methods for estimating mutual information are reviewed in Khan et al. (2007). In the calculations presented here, we used the algorithm constructed in Cellucci et al. (2005). This is a computationally efficient procedure. In test calculations it requires 0.5 % of the computation time required by the Fraser–Swinney algorithm (comparison calculations reported in Cellucci et al. 2005). Also, in contrast with other algorithms, the Cellucci algorithm incorporates an explicit calculation of the probability of the null hypothesis of no predictive relationship between the two variables. This statistical validation is particularly important in calculations with noisy psychophysiological data.

**Fig. 1:**
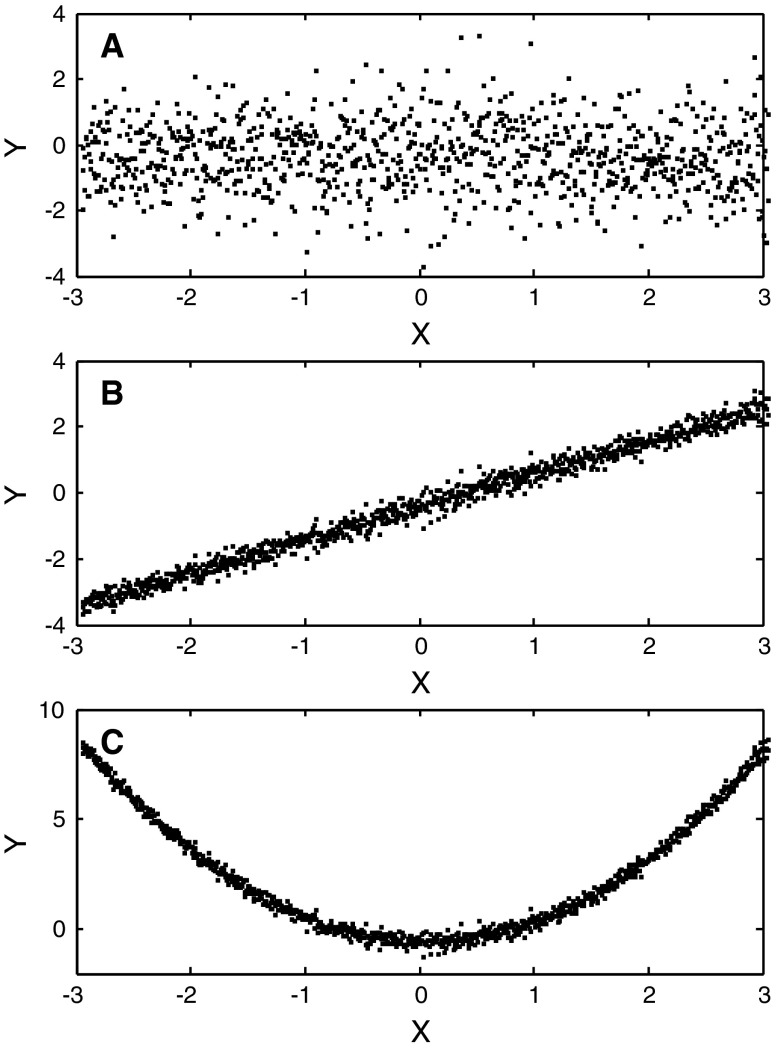
Three test signals used in the calculations reported in Table 2. In all cases x = −3 to +3 in steps of 0.0006. **a** Normally distributed random numbers with zero mean and unit variance. **b** y = x + 0.2 × ε, where ε is the first test signal. **c** y = x^2^ + 0.2 × ε. Ten thousand points were used in the calculations. Every tenth point is *plotted* on the diagram (modified from Cellucci et al. 2005)

An important property of mutual information is identified by examining the computational results presented in Fig. 1 and in Table 2 (modified from Cellucci et al. 2005 following an example in Mars and Lopes da Silva 1987). The first test signal consists of normally distributed random numbers. With each measure, the probability of the null hypothesis is significantly greater than zero. That is, each measure correctly failed to detect a nonrandom relationship between variables X and Y. In the case of linearly correlated signals each measure reports a P_NULL_ that is numerically indistinguishable from zero. Again, this is as it should be. An important distinction between measures is seen when the third signal, which is parabolically correlated, is examined. The Pearson product moment correlation failed to detect a linear correlation, P_NULL_ = 0.9912. The Spearman and Kendall measures which can identify monotonic nonlinear relationships also failed to reject the null hypothesis; P_NULL_ = 0.9928 and P_NULL_ = 0.9989 respectively. In contrast, mutual information identified a nonrandom relationship in parabolic data. The reported probability is of null hypothesis is indistinguishable from zero.

**Table 2:** Correlation calculations (modified from Cellucci et al. 2005)

	Normally distributed random	Linearly correlated	Parabolically correlated
Pearson r	r = −0.0037	r = 0.9934	r = 0.0001
Pearson P_NULL_	P_NULL_ = 0.7112	P_NULL_ ≈ 0	P_NULL_ = 0.9912
Spearman ρ_S_	ρ_S_ = −0.0040	ρ_S_ = 0.9936	ρ_S_ ≤ 10^−4^
Spearman P_NULL_	P_NULL_ = 0.6854	P_NULL_ ≈ 0	P_NULL_ = 0.9928
Kendall τ	τ = 0.0027	τ = 0.9270	τ ≤ 10^−5^
Kendall P_NULL_	P_NULL_ = 0.6845	P_NULL_ ≈ 0	P_NULL_ ≈ 0.9989
Mutual information (bits)	I = 0.1356	I = 2.9186	I = 3.0304
Mutual information P_NULL_	P_NULL_ = 0.7851	P_NULL_ ≈ 0	P_NULL_ ≈ 0

**Fig. 2:**
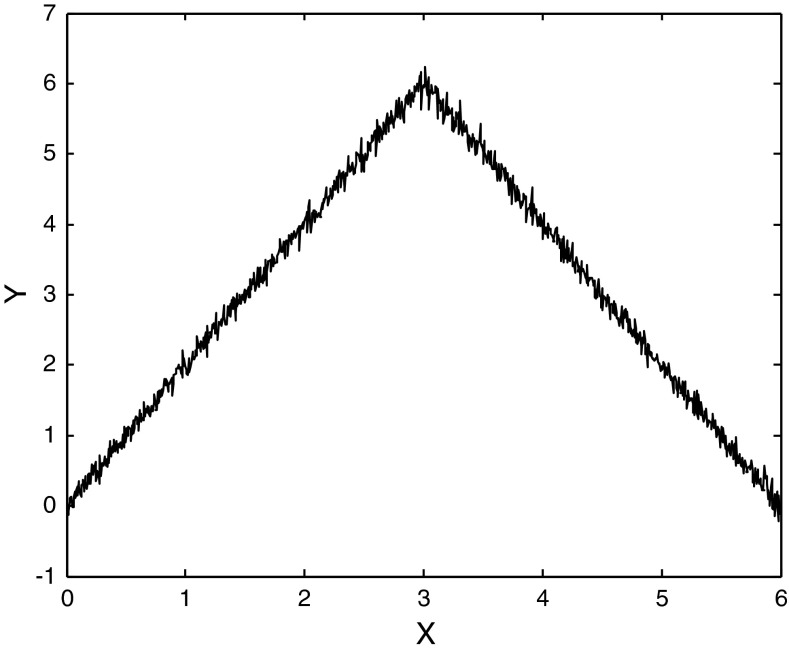
Non-monotonically correlated test signals. X = 0 to 6 in steps of 0.0006. Y = 2X + 0.1ε for X ∈ [0, 3] and Y = 12 − 2X + 0.1ε for X ∈ [3, 6]. All four measures detect a correlation for X ∈ [0, 3]. Only mutual information detects a nonrandom relationship when the paired signals are analyzed for X ∈ [0, 6]. Ten thousand points were used in the calculations. Every tenth point is *plotted* on the diagram

An additional lesson can be learned by considering the example shown in Fig. 2. In this system of paired signals X = 0–6 in steps of 0.0006 and

$$ {\text{Y}} = \left\{ {\begin{array}{*{20}c} {2{\text{X}} + 0.1 \times \varepsilon \quad 0 \le {\text{X}} \le 3} \hfill \\ {12 - 2{\text{X}} + 0.1 \times \varepsilon \quad 3 < {\text{X}} \le 6} \hfill \\ \end{array} } \right. $$

where, as before, ε is normally distributed with zero mean and unit variance. If the signals are examined over the first half of the diagram, X ∈ [0, 3], all four measures detect a significant relationship. P_NULL_ is numerically indistinguishable from zero in all four cases. If one considers X ∈ [0, 6], then the Pearson product moment correlation, Spearman rank order correlation and Kendall rank order correlation fail to reject the null hypothesis. For these measures, P_NULL_ is 0.959, 0.964 and 0.944 respectively. Mutual information, however, continues to identify a nonrandom relationship and P_NULL_ remains zero. Thus in the case of the three classical measures of correlation we have the seemingly paradoxical result that evidence for a relationship is lost as more data are available.

Two conclusions follow from the examples considered here. (1) Nonlinear measures should be used in combination with linear and nonparametric measures. (2) Evidence for time domain correlation should be examined as a function of epoch duration.

## Electroencephalographic data

The University’s Institutional Review Board reviewed and approved all procedures involving human subjects. Informed consents were obtained from each participant. There were thirteen participants. Participants were healthy adults without a history of head injury or serious psychiatric illness. Multichannel monopolar recordings, referenced to linked earlobes, were obtained from F_Z_, C_Z_, P_Z_, O_Z_, F_3_, F_4_, C_3_, C_4_, P_3_, and P_4_ using an Electrocap and Sensorium EPA-6 amplifiers. Vertical and horizontal eye movements were recorded from electrode sites above and below the right eye and from near the outer canthi of each eye. Artifact corrupted records were removed from the analyses. Artifact corruption was defined as an amplitude difference greater than 120 μV peak-to-peak within 500 msec or a blink in the EOG channel. All EEG impedances were less than 5 KOhm. Signals were amplified, Gain = 18,000, and amplifier frequency cutoff settings of 0.03 and 200 Hz were used. Signals were digitized at 1,024 Hz using a twelve-bit digitizer. Multichannel records were obtained in two conditions: eyes closed, resting and eyes open, resting. Continuous artifact-free records were obtained from each subject in the two conditions (eyes open and eyes closed). Given the results shown in Fig. 2, measures were calculated as a function of epoch duration (1–8 s).

## Comparing measures in between-state discriminations

The psychophysiological utility of each measure was assessed by determining its ability to discriminate between eyes open, no task and eyes closed, no task conditions. For concreteness of presentation, the experiment is described by considering the first measure, the product moment correlation which is denoted by r. The EEGs are ten-channel recordings. Thus for a single participant there are 45 distinct channel pairs. The correlation between channel i and channel j, r_ij_, is measured in each condition to give 45 values of (r_ij_)_closed_ and 45 values of (r_ij_)_open_. The operational question becomes can we discriminate between states by comparing (r_ij_)_closed_ against (r_ij_)_open_? As noted above, there were thirteen participants in the study. This gives 585 (number of participants × number of channel pairs) (r_ij_)_closed_ versus (r_ij_)_open_ pairs. They are compared in a paired *t* test. The test produces a value of t and the corresponding probability of the null hypothesis. In this application the null hypothesis supposes that there is no difference in between-channel correlations in the eyes open and eyes closed correlation. A high value of t, and hence a low value of P_NULL_, indicates a successful discrimination.

This process is performed for all four measures. As operationalized in this study, the comparative assessment of these measures of correlation can now be stated in a single question. Which measure gives the largest value of t and lowest values of P_NULL_? Concerns have been expressed (Gevins 1987) about the amount of data required to estimate mutual information. The calculations have, therefore, been repeated for 1, 2, …, 8 s epochs.

**Fig. 3:**
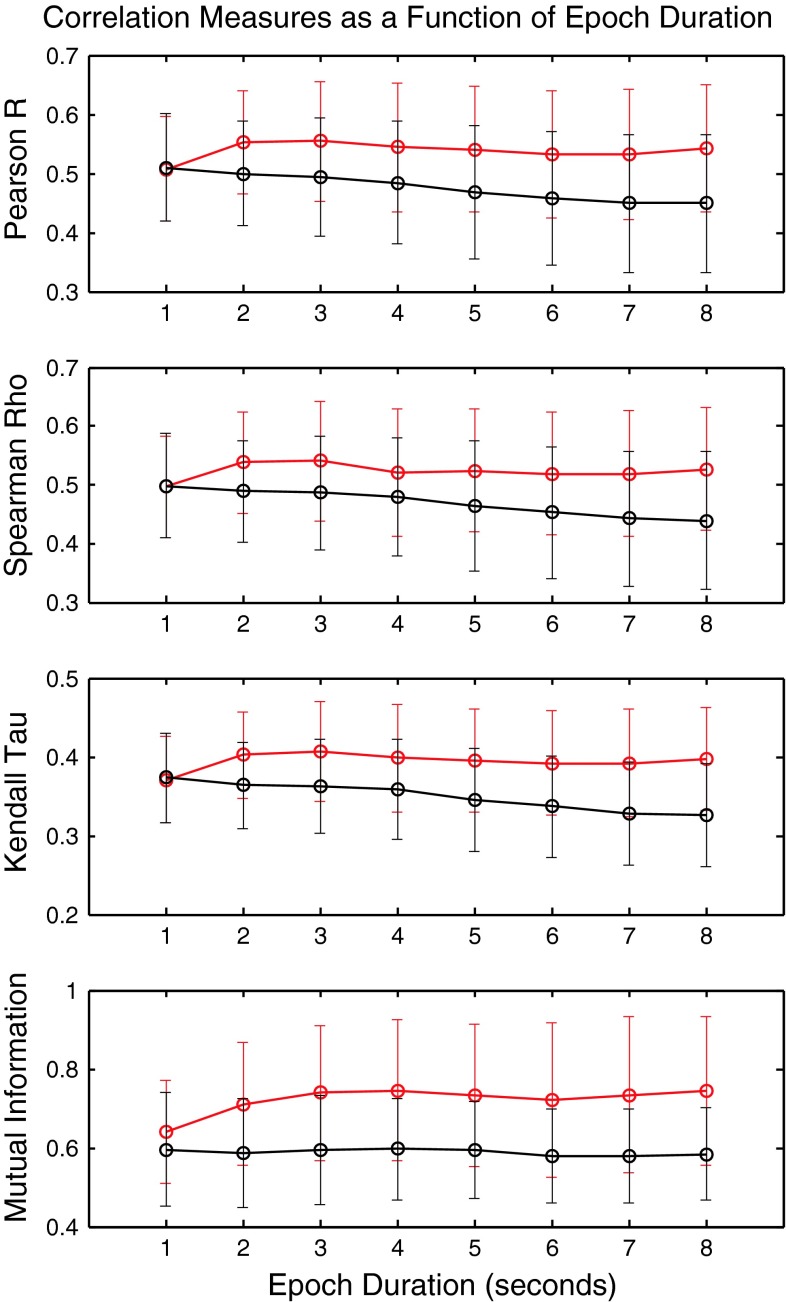
Correlation measures as a function of epoch length. The mean values of Pearson r, Spearman rho, Kendall tau and mutual information are calculated for the indicated epoch duration. Values in *red* are group means and standard deviations for the eyes-closed condition. Values in *black* were obtained with eyes-open data

The values of these four measures are shown in Fig. 3. The results are consistent with expectations. There is a greater between-channel correlation (Pearson, Spearman, Kendall) in the eyes closed condition. Similarly, there is a greater between-channel predictability (mutual information) in the eyes closed condition.

**Fig. 4:**
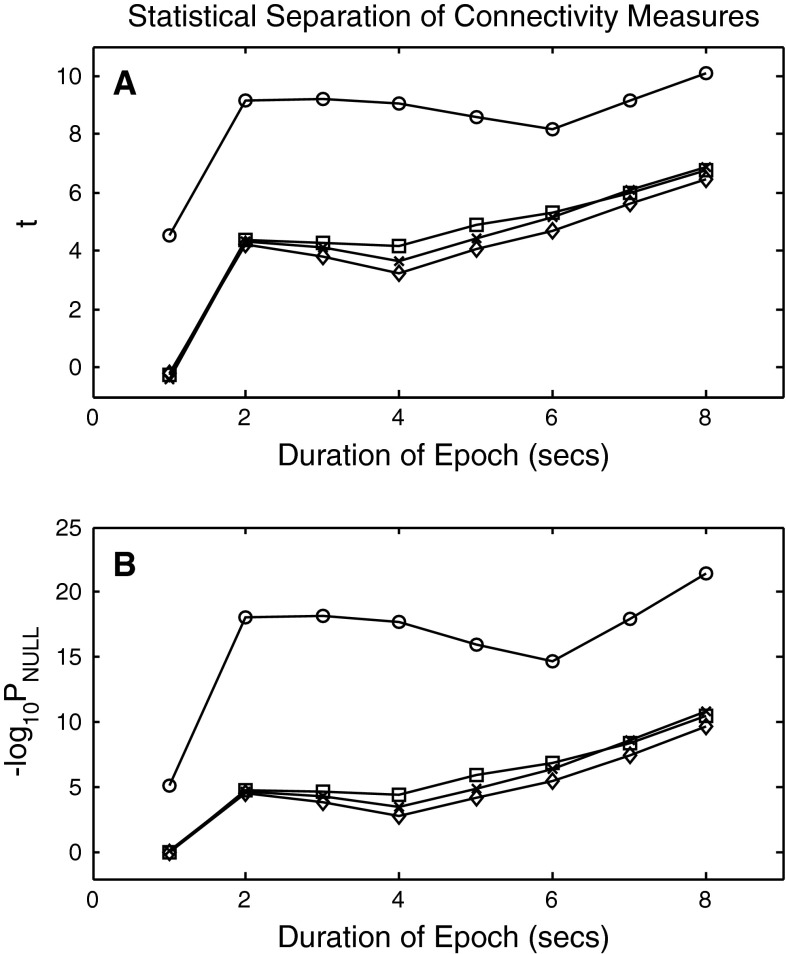
Comparison of correlation measures as a function of epoch length. **a** Values of t obtained in an eyes closed versus eyes open paired *t* test as a function of epoch duration. **b** Values of −log_10_ P_NULL_ for the corresponding probabilities of the null hypothesis. **a**, **b** *Squares* identify results from the Pearson product moment correlation. *Diamonds* identify results from the Spearman rank order correlation. The *letter x* identifies results from the Kendall rank order correlation and *circles* identify results obtained with mutual information

The uncertainties shown in Fig. 3 are standard deviations of group means. When comparing correlation results obtained in the eyes-closed condition against those in the eyes open condition the appropriate comparison is not based on group means and standard deviations. Rather, the comparison is by matched channel pairs. For example, the C3–C4 correlation observed in the eyes-closed condition is compared against the C3–C4 correlation obtained in the eyes-open condition. The collective statistical result of this paired test is shown in Fig. 4. The upper panel shows the t values obtained in the eyes open versus eyes closed paired *t* test for epoch durations of 1, 2, …, 8 s. In the case of 1 s durations, Pearson, Spearman and Kendall correlations do not discriminate between the two behavioral conditions. They fail to reject the null hypothesis. The respective values of P_NULL_ are 0.807, 0.854 and 0.699. The null hypothesis is, however, rejected for 1 s durations by mutual information where P_NULL_ <10^−5^. All four measures reject the null hypothesis at epoch durations greater than or equal to 2 s. In all cases, the value of t obtained with mutual information is greater than the value obtained with the other measures. A further understanding of the between state discrimination can be obtained by examining the restatement of the results that is given in the second panel of the diagram where −log_10_ (P_NULL_) is plotted as a function of epoch duration. A value of +5, for example, on this graph corresponds to P_NULL_ = 10^−5^ The values of −log_10_ (P_NULL_) obtained with mutual information are consistently greater than those obtained with the other measures.

## Robustness to noise

**Fig. 5:**
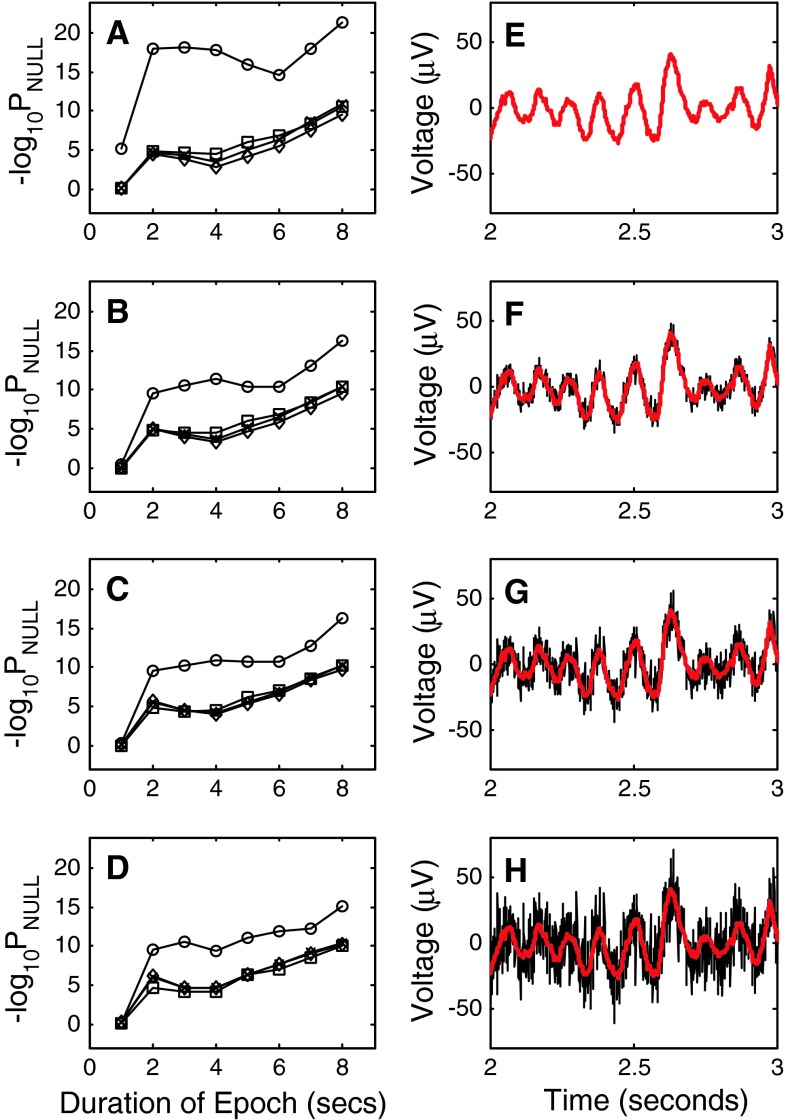
Robustness of correlation measures to additive gaussian noise. **a** Comparison of correlation measures using original data from 13 subjects. As before, *squares* identify results from the Pearson product moment correlation. *Diamonds* identify results from the Spearman rank order correlation. The *letter x* identifies results from the Kendall rank order correlation and *circles* identify results obtained with mutual information. **b** Comparison of correlation measures using data from 13 subjects following addition of gaussian noise giving signal to noise ratios of SNR = 10 dB. *Symbols* identifying different measures follow the pattern of **a**. **c** Comparison of correlation measures using data from 13 subjects following addition of gaussian noise giving signal to noise ratios of SNR = 5 dB. *Symbols* identifying different measures follow the pattern of **a**. **d**. Comparison of correlation measures using data from 13 subjects following addition of gaussian noise giving signal to noise ratios of SNR = 0 dB. *Symbols* identifying different measures follow the pattern of **a**. **e** Example segment of an EEG signal recorded from a single subject at electrode site P_z_ in the eyes closed condition. **f.** Component of the EEG signal shown in **e** after addition of gaussian noise, SNR = 10 dB (shown in *black*). The original signal is shown in *red* for comparison. **g** Component of the EEG signal shown in **e** after addition of gaussian noise, SNR = 5 dB (shown in *black*). The original signal is shown in *red* for comparison. **h** Component of the EEG signal shown in **e** after addition of gaussian noise, SNR = 0 dB (shown in *black*). The original signal is shown in *red* for comparison

Gevins (1987) raised questions concerning the sensitivity of mutual information calculations to noise. Notably, he did so in the context of the Callaway and Harris (1974) study where the voltage time series were encoded by polarity and sign of the derivative. We have investigated noise sensitivity in the case of direct voltage time series calculations by testing the robustness of these measures to additive noise. All four measures were found to be robust to noise, but as in the previous calculations, mutual information outperformed the other three measures. In this experiment, normally distributed random numbers with zero mean were added to each of the original EEG signals. The random number generator was based on Park and Miller (1988) and incorporated a Bays–Durham shuffle (Knuth 1981) followed by a Box–Muller transformation (Press et al. 1992). The variance of the additive noise was progressively increased to give signal to noise ratios of 10, 5 and 0 dB. A qualitative understanding of each signal to noise ratio is given in Panels F, G and H of Fig. 5. The signal presented in black is the noise corrupted signal. This is the input signal used in the calculations. The red signal is the original signal. For reference, it is superimposed on the corrupted signal.

At SNR = 10 dB all four measures failed to discriminate between conditions when 1 s epochs were examined. All four measures successfully made the discrimination for greater epoch lengths, but as in the case of uncorrupted signals, a greater statistical separation was obtained with mutual information.

At higher noise levels (lower SNR) the degree of between state discrimination as quantified by P_NULL_ is reduced, but the pattern observed with SNR = 10 dB is preserved. Specifically, all four measures fail to discriminate between eyes closed and eyes open with 1 s epochs. All four measures successfully discriminate at longer epochs, and the degree of discrimination obtained with mutual information is greater than that observed with the other three measures.

## Discussion

Three results were obtained in these calculations. First, a nonlinear measure, mutual information, effectively discriminated between states with less data, specifically a 1 s epoch, when other measures failed to discriminate between conditions. Second, at all epoch durations tested, the measure of between-state discrimination was greater for mutual information. Third, discrimination based on mutual information was more robust to noise.

The limitations of this study should be recognized. Three points should be addressed. First, the study is based on signals obtained from thirteen participants. Because the method that is best for one database is not necessarily best in all cases, a different outcome may be obtained with different data. Second, in this study the test criterion was the ability to discriminate between the eyes-open and eyes-closed condition. It is possible that a different measure, a measure other than mutual information, would be more effective if a different test criterion was implemented. Third, this study was limited to a comparison of four time domain measures of correlation. Several other measures have been used to quantify correlation and should be considered. Reshef et al. (2011) have constructed a maximal information criterion that has some properties in common with mutual information. Additional methods include coherence (Nunez et al. 1997, 1999), phase locking index (Stam et al. 2009; Hurtado et al. 2004; Sazonov et al. 2009), imaginary coherency (Stam et al. 2007a, b; Nolte et al. 2004) and phase lag index (Stam et al. 2007a, b, 2009). As outlined by several authors (Cao and Slobounov 2010; Schiff 2005; Guevara et al. 2005), care must be exercised in the application of these procedures. Recently more sophisticated procedures for assessing correlation have been investigated. Stam and van Djik (2002) and Wendling et al. (2009) have used methods based on embedded data (Takens 1981) to quantify correlation. Cao and Slobounov (2010) analyzed nineteen channel resting EEGs in a three step process. First, independent component analysis (Hyvärinen et al. 2001) was used to identify independent processes. Second, a source reconstruction algorithm (standardized low resolution electromagnetic tomography, sLORETA (Pascual-Marqui et al. 2002; Pascual-Marqui 2002) was used to identify cortical regions associated with functional activity. Third, using this localization, graph theory was used to quantify connectivity in the resting state. These procedures should be incorporated into an expanded comparison study. The Wendling et al. (2009) results obtained with computationally generated data indicated that no single procedure was best for all cases. This is almost certainly true for biological data. The importance of using more than one measure was further indicated by the results of Dauwels et al. (2010) who found that different measures of synchronization were not well correlated. They concluded that “therefore they each seem to capture a specific kind of interdependence.” Our best recommendation is to perform functional connectivity studies with several methods including both original scalp signals and estimates of current source density and compare the results.

It is possible to use mutual information calculations in synchronization studies. In this experimental design, the original EEG signal is bandpass filtered into specified frequency bands. Given the restricted spectrum of the filtered signal, it is possible to estimate its phase by calculating the Hilbert transform (Boashash 1992; Pikovsky et al. 2001). Mutual information calculations can then determine if there is a nonrandom relationship between phase functions measured at different electrode sites.

While recognizing the limitations of this study, the results suggest that when implemented with an adaptive partition of the joint probability distribution, mutual information provides an effective noise-robust measure of correlation. This result may extend beyond functional connectivity studies to include analysis of CNS causal networks and analysis of CNS small world networks, which are briefly considered.

Investigation of CNS causal relationships, the time dependent directional movement of information, may be important in the study of traumatic brain injury. As previously noted, Goldstein’s pioneering work on the behavioral neurology of traumatic brain injury led him to conclude that restitution of function following injury resulted from adaptation rather than from repair. This suggests that post-injury alteration of causal networks may provide a sensitive measure of altered CNS function following injury. While measures like correlation, coherence and mutual information can be used to establish the presence of correlative relationships between signals they do not provide any information about the direction of information movement. Additional procedures must be introduced. In most cases, the quantitative assessment of causal relationships between variables is constructed on the following idea. If measuring variable X improves the prediction of variable Y, then Y is, in this limited operational sense, causally dependent on X. It should be stressed that this relationship is not necessarily unidirectional. It can also be the case that with the same data, measuring Y also improves the prediction of X. This conceptualization of causality appears in Wiener (1956) and may be original with Wiener.

An early implementation of this operationalization of causality was published by Granger (1969) in the econometrics literature and popularized by Sims (1972). Granger causality is constructed using linear regression models. If past values of X are useful in predicting the current value of Y in a linear regression, then X is said to be a causal drive of time series Y. As with any statistical procedure, causality tests based on linear regression must be implemented with care. A growing literature has identified circumstances that lead to spurious identification of linear causality (Breitung and Swanson 2002; He and Maekawa 2001).

An extension of mutual information may provide a noise-robust measure of causality. Recall that the mutual information of time series X and Y, I(X, Y) is the average number of bits of one variable that can be predicted by measuring the other. Mutual information can be shown to be symmetrical, that is I(X, Y) = I(Y, X). Therefore while mutual information can establish the presence of a nonrandom relationship between time series, it cannot identify causal relationships. However, a time lagged mutual information in which one of the two variables is time shifted can be used to determine, if, for example, measuring variable X in the past allows prediction of future values of variable Y. We can shift time series X by lag τ and calculate I(X_τ_, Y) as a function of τ. Similarly, we can calculate I(X, Y_τ_). If measuring X_τ_ allows better prediction of Y, than the other way around, then it can be argued that information is transferred from X to Y. The magnitude of the mutual information and the time lag which produces the greatest value can be used to quantify both the magnitude of the information transfer and the time delay associated with that transfer. A number of investigators have proposed using lagged mutual information to investigate information transfer in distributed systems (Kaneko 1986; Vastano and Swinney 1988; Albano et al. 1999). The procedure has a long history in electroencephalography. Inouye et al. (1983) used an “entropy analysis” which was what would now be described as directed mutual information to quantify the direction of information flow and concluded that the dominant longitudinal direction of alpha activity was anterior to posterior. A subsequent publication (Inouye et al. 1993) used directed mutual information to show change in information flow during a cognitively demanding arithmetic task. Mars and his colleagues (Mars and Lopes da Silva 1983; Mars et al. 1985) used mutual information to quantify time delays in the transmission of epileptic seizures. Several other investigators have used lagged mutual information to quantify between-channel information transfer in multichannel EEGs (Xu et al. 1997; Chen et al. 2000; Lopes da Silva 1987). Schreiber (2000), however, has presented valuable results which produced examples where standard lagged mutual information failed to detect information exchange. This motivated the construction of a related measure, transfer entropy, that successfully identified these relationships. The Schreiber results should be considered in the light of the previously cited Duckrow and Albano (2003) calculations that demonstrated the sensitivity of mutual information calculations on the choice of algorithm. This may have been a factor in the Schreiber study. Madulara et al. (2012) calculated transfer entropy using the EEG records analyzed in this paper. Mutual information was generally lower in the eyes open than in the eyes closed condition. In contrast, transfer entropies increased by a factor of two in the eyes open condition. As would be anticipated, the largest one-way transfer entropies were observed to and from the occipital lobe. Consistent with our previous recommendations, we suggest computing both measures (lagged mutual information and transfer entropy). Clinical utility is the final arbiter.

Stated in abstract terms a network is a collection of nodes and connections between the nodes. A small world network is defined as a network that has dense local clusters that are connected by a limited number of long range connections. In a seminal paper, Watts and Strogatz (1998) showed how small world networks can be characterized quantitatively. Small world networks are highly efficient. They can support a high degree of dynamical complexity with a minimum investment in connections (Latora and Marchiori 2001). This is an attractive metaphor for describing the central nervous system. Local networks provide areas of specialization, but these specialized domains can communicate efficiently with the entire brain by long range connections. When applied to multichannel EEG data, the electrode sites are the nodes and the connections are identified by correlation measures. Three types of connections can be identified. In a binary network, a connection is either present or absent. Operationally this is established by assigning a threshold value (connection present/absent) to a measure of correlation. In a weighted network, the value of a connection’s strength is assigned on a continuum determined by the correlation measure. In directed networks, the direction of information transfer, not just the strength of the connection, is incorporated into the analysis. These methods are now being utilized in the analysis of the central nervous system (Smith-Bassett and Bullmore 2006; Sporns and Honey 2006; Stam and Reijneveld 2007). Altered small world networks have been observed in clinical populations including patients with CNS tumors (Bartolomei et al. 2006), epilepsy (Ponten et al. 2007; van Dellen et al. 2009), schizophrenia (Rubinov et al. 2009), and Alzheimer’s disease (Stam et al. 2007a, b). As would be anticipated alterations in networks are associated with traumatic brain injury (Cao and Slobounov 2010; Nakamura et al. 2009; Tsirka et al. 2011; Zouridakis et al. 2011; Catsellanos et al. 2011a, b). The calculations presented in this paper and in Madulara et al. (2012) suggest that when calculated using an adaptive partition of the joint probability distribution, mutual information, lagged mutual information and transfer entropy can provide computationally efficient, noise-robust metrics for the analysis of CNS small world networks.

The mathematical results showing the efficiency of networks composed of highly connected local regions with limited, but essential, long range connections can inform the discussion of CNS localization of function. The localizationist conceptualization began with Broca’s localization of expressive aphasia to the third left frontal convolution (Broca 1861) and Wernicke’s localization of receptive aphasia to the posterior section of the superior temporal convolution (Wernicke 1908). By the early twentieth century, however, several neurologists argued against a strict localizationist model (Tesak and Code 2008). Kurt Goldstein was a significant contributor to the debate (Goldstein 1927; Ludwig 2012). Goldstein’s views were complex and it would be an oversimplification to describe his views as inflexibly antilocalizationist (Ludwig 2012). For example, in his Lokalisation in der Großhirnrinde, Goldstein recognizes Broca’s “flawless establishment of the dependency of the impairment of articulated speech from a lesion in the third left frontal convolution” (Goldstein 1927, translated Ludwig 2012). He similarly accepts Wernicke’s identification of the role of the superior temporal convolution in some presentations of receptive aphasia, but based on clinical observations Goldstein concluded that language functions could not be decomposed into discrete anatomically isolated components. Goldstein’s acceptance of localizationist results but his argument for the incompleteness of a localizationist account caused Geschwind (1997) to describe his views as a “paradoxical position.” Ludwig proposes that the paradox can be resolved by recognizing that Goldstein introduced a distinction between weak localization (the correlation of symptoms with lesions) and strong localization (the implementation of a process exclusively in a defined locality). We suggest that a quantitative examination of these questions can be constructed by comparing CNS network geometries generated by language dependent ERP tasks in healthy controls and in patients presenting well characterized aphasias.

## Appendix: Measures of correlation

### Pearson product moment correlation

Let $\{ {\text{X}}\} = \{ {\text{x}}_{1} ,{\text{x}}_{2} , \ldots ,{\text{x}}_{{{\text{N}}_{\text{D}} }} \}$ and $\{ {\text{Y}}\} = \{ {\text{y}}_{1} ,{\text{y}}_{2} , \ldots ,{\text{y}}_{{{\text{N}}_{\text{D}} }} \}$ be time series of paired observations. N_D_ is the number of elements in each set. The product moment correlation coefficient r is given by

$$ {\text{r}} = \frac{{\sum\nolimits_{{{\text{i}} = 1}}^{{{\text{N}}_{\text{D}} }} {({\text{x}}_{\text{i}} - \overline{\text{x}} )({\text{y}}_{\text{i}} - \overline{\text{y}} )} }}{{\left\{ {\sum\nolimits_{{{\text{i}} = 1}}^{{{\text{N}}_{\text{D}} }} {({\text{x}}_{\text{i}} - \overline{\text{x}} )^{2} } } \right\}^{1/2} \left\{ {\sum\nolimits_{{{\text{i}} = 1}}^{{{\text{N}}_{\text{D}} }} {({\text{y}}_{\text{i}} - \overline{\text{y}} )^{2} } } \right\}^{1/2} }} $$

where $\overline{\text{x}}$ is the mean of {X} and $\overline{\text{y}}$ is the mean of {Y}. There are several procedures for calculating the probability of the null hypothesis of no correlation between {X} and {Y} (Press et al. 1992). A robust procedure that was used here is based on a t-distribution where

$$ {\text{t}} = {\text{r}}\left\{ {\frac{{{\text{N}}_{\text{D}} - 2}}{{1 - {\text{r}}^{2} }}} \right\}^{1/2} $$

and ν = N_D_ − 2 is the number of degrees of freedom., The probability of the null hypothesis is

$$ {\text{P}}_{\text{NULL}} = I_{{\frac{\upnu }{{\upnu + {\text{t}}^{2} }}}} \left( {\frac{\upnu }{2},\frac{1}{2}} \right). $$

I_x_(a, b) is the incomplete beta function.

The 95 % confidence limits for r, r_Low_ and r_High_, can be computed by converting r to Fisher’s z.

$$ {\text{z}} = \frac{1}{2}\ln \left\{ {\frac{{1 + {\text{r}}}}{{1 - {\text{r}}}}} \right\} $$

Z is normally distributed and has a standard deviation $\upsigma = 1/({\text{N}}_{\text{D}} - 3)^{1/2}$ (Press et al. 1992, p. 632). The 95 % confidence bounds are ${\text{z}}_{\text{Low}} = 1 - 1.96\upsigma$ and ${\text{z}}_{\text{High}} = 1 + 1.96\upsigma$. The corresponding values of r are found from

$$ {\text{r}} = \frac{{{\text{e}}^{{2{\text{z}}}} - 1}}{{{\text{e}}^{{2{\text{z}}}} + 1}} $$

Press et al. (1992, p. 631) suggest that this should be a legitimate calculation for N_D_ > 10.

### Spearman rank order correlation

As before let {X} and {Y} be time series of N_D_ paired observations. $\{ {\text{R}}_{\text{X}} \} = \left\{ {{\text{R}}_{{{\text{X}}_{1} }} ,{\text{R}}_{{{\text{X}}_{2} }} , \ldots ,{\text{R}}_{{{\text{X}}_{{{\text{N}}_{\text{D}} }} }} } \right\}$ gives the ranks of the values of X. In cases of ties the average ranks are entered. {R_Y_} is defined analogously. The Spearman rank order correlation, ρ_S_, is the product moment correlation of ranks.

$$ \uprho_{\text{S}} = \frac{{\sum\nolimits_{{{\text{i}} = 1}}^{{{\text{N}}_{\text{D}} }} {\left( {{\text{R}}_{{{\text{X}}_{\text{i}} }} - \overline{\text{R}}_{\text{X}} } \right)\left( {{\text{R}}_{{{\text{Y}}_{\text{i}} }} - \overline{\text{R}}_{\text{Y}} } \right)} }}{{\left\{ {\sum\nolimits_{{{\text{i}} = 1}}^{{{\text{N}}_{\text{D}} }} {\left( {{\text{R}}_{{{\text{X}}_{\text{i}} }} - \overline{\text{R}}_{\text{X}} } \right)} } \right\}^{1/2} \left\{ {\sum\nolimits_{{{\text{i}} = 1}}^{{{\text{N}}_{\text{D}} }} {\left( {{\text{R}}_{{{\text{Y}}_{\text{i}} }} - \overline{\text{R}}_{\text{Y}} } \right)} } \right\}^{1/2} }} $$

When there are no ties this becomes

$$ \uprho_{\text{S}} = 1 - \frac{{6\sum\nolimits_{{{\text{i}} = 1}}^{{{\text{N}}_{\text{D}} }} {\left( {{\text{R}}_{{{\text{X}}_{\text{i}} }} - {\text{R}}_{{{\text{Y}}_{\text{i}} }} } \right)^{2} } }}{{{\text{N}}_{\text{D}}^{3} - {\text{N}}_{\text{D}} }} $$

It can be shown that ρ_S_ reduces to the Pearson product moment correlation when calculations are performed on ranks in the absence of ties. The probability of the null hypothesis (no correlation) is calculated as before with t taking the value t_S_.

$$ {\text{t}}_{\text{S}} = \uprho_{\text{S}} \left\{ {\frac{{{\text{N}}_{\text{D}} - 2}}{{1 - \uprho_{\text{S}}^{2} }}} \right\}^{1/2} $$

The Spearman rank order correlation is less sensitive to outliers than the product moment correlation. Importantly, the rank order correlation can detect nonlinear correlations provided that the relation between X and Y is approximately monotonic. A helpful example is given in Triola (2008, p. 713). The rank order correlation is less efficient than the product moment correlation in the sense that the nonparametric measure requires 100 observations to achieve the same results as 91 observations analyzed with the Pearson correlation (Triola 2008, p. 677).

### Kendall rank order correlation

Consider two consecutive paired observations (X_i_, Y_i_) and (X_i+1_, Y_i+1_). If both X and Y increase, then X_i+1_−X_i_, Y_i+1_−Y_i_, and (X_i+1_−X_i_)(Y_i+1_−Y_i_) are positive. If both variables decrease between observation i and i + 1, then (X_i+1_−X_i_)(Y_i+1_−Y_i_) is again positive. If these two variables are negatively correlated between these two observations, then (X_i+1_−X_i_)(Y_i+1_−Y_i_) is negative. The Kendall rank correlation coefficient is constructed by examining these relationships over all possible pairs of observations. If (X_i+1_−X_i_)(Y_j+1_−Y_j_) is positive, then variable κ is increased by 1. If (X_i+1_−X_i_)(Y_i+1_−Y_i_) is negative then variable κ is decreased by 1. If it is zero, then κ is unchanged. These comparisons are made not just across temporally adjacent pairs, that is between (X_i_, Y_i_) and (X_i+1_, Y_i+1_), but rather for all possible (X_i_, Y_i_) and (X_j_, Y_j_) pairs. There are N_D_(N_D_ − 1)/2 distinct pairs, giving κ a maximum possible value of N_D_(N_D_ − 1)/2. Kendall’s τ is the normalized value of κ.

$$ \uptau = \frac{\upkappa }{{{\text{N}}_{\text{D}} ({\text{N}}_{\text{D}} - 1)/2}} $$

τ has a value between −1 and +1. In the null hypothesis of no association between {X} and {Y}, τ is normally distributed and has the standard deviation (Press et al. 1992, p. 637).

$$ \upsigma = \left\{ {\frac{{4{\text{N}}_{\text{D}} + 10}}{{9{\text{N}}_{\text{D}} ({\text{N}}_{\text{D}} - 1)}}} \right\}^{1/2} $$

The probability of the null hypothesis is computed from the complementary error function.

$$ {\text{P}}_{\text{NULL}} = \frac{2}{\sqrt \uppi }\int\limits_{|\uptau |/\sqrt 2 \upsigma }^{\infty } {{\text{e}}^{{ - {\text{t}}^{2} }} {\text{dt}}} $$

Again letting ${\text{R}}_{{{\text{X}}_{\text{i}} }}$ and ${\text{R}}_{{{\text{Y}}_{\text{i}} }}$ denote the ranks of {X} and {Y}, it is seen that $({\text{R}}_{{{\text{X}}_{\text{i}} }} - {\text{R}}_{{{\text{X}}_{\text{j}} }} )({\text{R}}_{{{\text{Y}}_{\text{i}} }} - {\text{R}}_{{{\text{Y}}_{\text{j}} }} )$ has the same sign as (X_i_ − X_j_)(Y_i_ − Y_j_) and therefore κ calculated from ranks is identical to κ calculated using X and Y values. τ is therefore seen to be a nonparametric correlation that does not make any assumptions about the distributions of {X} and {Y}. It is generally observed that ρ_S_ and τ are highly correlated. This anticipation is borne out in the calculations presented here. τ provides a means of identifying monotonic correlations. A more general search for correlations which would include non-monotonic associations requires alternative measures.

### Mutual information

Given {X} and {Y}, time series of paired observations. Again, N_D_ is the number of elements in each set. The mutual information of variables X and Y, denoted I(X, Y), is the average number of bits of variable Y that can be predicted by measuring variable X. It can be shown (Cover and Thomas 1991) that mutual information is symmetrical; I(X, Y) = I(Y, X). For finite data sets I(X, Y) can be approximated by estimating the probability distributions of each variable and their joint probability distribution. Each variable’s distribution is approximated by a histogram. Let N_X_ be the number of bins in the histogram of variable X. O_X_(i) is the occupancy of the i-th bin and P_X_(i) = O_X_(i)/N_D_ is the probability of occupation in the i-th bin. (The procedure for determining N_X_ and the upper and lower bound of each element of the partition is described presently.) N_Y_ is the number of elements in the histogram of variable Y. In the general case N_X_ and N_Y_ are not necessarily equal. O_Y_(i) and P_Y_(i), j = 1, 2, …, N_Y_ are the corresponding occupancies and probabilities.

P_XY_(i, j), the joint probability distribution, is the probability that an (x, y) pair is an element in the i-th bin of the partition of the X axis and the j-th bin of the partition of the Y axis. Mutual information is defined by

$$ {\text{I}}({\text{X}},{\text{Y}}) = \sum\limits_{{{\text{i}} = 1}}^{{{\text{N}}_{\text{X}} }} {\sum\limits_{{{\text{j}} = 1}}^{{{\text{N}}_{\text{Y}} }} {{\text{P}}_{\text{XY}} ({\text{i}},{\text{j}})} } \log_{2} \left\{ {\frac{{{\text{P}}_{\text{XY}} ({\text{i}},{\text{j}})}}{{{\text{P}}_{\text{X}} ({\text{i}}){\text{P}}_{\text{Y}} ({\text{j}})}}} \right\} $$

where there is no contribution to the sum if P_XY_(i, j) = 0. If variables X and Y are statistically independent, then P_XY_(i, j) = P_X_(i)P_Y_(j) and I(X, Y) = 0. Thus in a calculation of mutual information, the null hypothesis is statistical independence of variables X and Y, in which case I(X, Y) is indistinguishable form zero. Let E_XY-NULL_(i, j) be the expected occupancy of element (i, j) of the XY partition if X and Y are independent. Under the assumption of independence E_XY-NULL_(i, j) becomes

$$ {\text{E}}_{{{\text{XY}} - {\text{NULL}}}} (i,j) = {\text{N}}_{\text{D}} {\text{P}}_{\text{X}} ({\text{i}}){\text{P}}_{\text{Y}} ( {\text{j)}} $$

Let O_XY_(i, j) be the observed occupancy in each element of the partition. The corresponding value of χ^2^ is

$$ \upchi^{2} = \sum\limits_{{{\text{i}} = 1}}^{{{\text{N}}_{\text{X}} }} {\sum\limits_{{{\text{j}} = 1}}^{{{\text{N}}_{\text{Y}} }} {\frac{{\{ {\text{E}}_{{{\text{XY}} - {\text{NULL}}}} ({\text{i}},{\text{j}}) - {\text{O}}_{\text{XY}} ({\text{i}},{\text{j}})\}^{2} }}{{{\text{E}}_{{{\text{XY}} - {\text{NULL}}}} ({\text{i}},{\text{j}})}}} } $$

The number of degrees of freedom, υ, is given by υ = (N_X_ − 1)(N_Y_ − 1). The probability of the null hypothesis of statistical independence is

$$ {\text{P}}_{\text{NULL}} = {\text{Q}}\left( {\frac{\upsilon }{2},\frac{{\upchi^{2} }}{2}} \right) $$

Q is the incomplete gamma function.

$$ {\text{Q}}({\text{a}},{\text{x}}) = \frac{1}{{\Upgamma ({\text{a}})}}\int\limits_{\text{x}}^{\infty } {{\text{e}}^{{ - {\text{t}}}} } {\text{t}}^{{{\text{a}} - 1}} {\text{dt}} $$

$$ \Upgamma (a) = \int\limits_{0}^{\infty } {{\text{e}}^{{ - {\text{t}}}} } {\text{t}}^{{{\text{a}} - 1}} {\text{dt}} $$

When examining finite data sets the estimated value of I(X, Y) is critically dependent on the partition of X and Y values used to estimate P_X_(i), P_Y_(i) and P_XY_(i, j). Several different procedures can be used to estimate these distributions. We apply here a specific implementation of an algorithm using a nonuniform partition that was introduced in Cellucci et al. (2005). This algorithm considers the special case where the same number of elements, N_E_, is used to partition the X and Y variables; N_E_ = N_X_ = N_Y_. The bins span the range x_min_ to x_max_ on the X axis and y_min_ to y_max_ on the Y axis. In this algorithm, the widths of the bins are varied independently on each axis to meet the criterion of uniform occupancy; that is each element has occupancy N_D_/N_E_ = O_X_(i) = O_Y_(j) giving P_X_(i) = P_Y_(j) = 1/N_E_. It should be understood, however, that the values of P_XY_(i, j) will not be uniform. The equi-probable partition of each axis ensures that

$$ \sum\limits_{{{\text{i}} = 1}}^{{{\text{N}}_{\text{E}} }} {{\text{P}}_{\text{XY}} ({\text{i}},{\text{j}}) = \sum\limits_{{{\text{j}} = 1}}^{{{\text{N}}_{\text{E}} }} {{\text{P}}_{\text{XY}} ({\text{i}},{\text{j}}) = 1/{\text{N}}_{\text{E}} } } $$

But the P_XY_(i, j) values will be different. The assumption of a partition giving P_X_(i) = P_Y_(j) = 1/N_E_ gives

$$ {\text{E}}_{{{\text{XY}} - {\text{NULL}}}} ({\text{i}},{\text{j}}) = {\text{N}}_{\text{D}} {\text{P}}_{\text{X}} ({\text{i}}){\text{P}}_{\text{Y}} ({\text{j}}) = {\text{N}}_{\text{D}} /{\text{N}}_{\text{E}}^{2} $$

When estimating P_XY_(i, j) we must address the question, what is the appropriate value of N_E_? This is the two dimensional analog of the histogram problem, which is the appropriate number of bins in a histogram? The morphology of the distribution cannot be detected if the number of bins is too small. This is seen by consider the limiting case of a single bin. Conversely, if the number of bins is too large, occupancies are zero or one and again the shape of the distribution cannot be determined. The number of bins for either a one dimensional or two dimensional distribution should be as large as possible, but not too large. In this algorithm, N_E_ is determined by applying a variant of the Cochran criterion (Cochran 1954) to E_XY-NULL_(i, j). This criterion requires E_XY-NULL_(i, j) ≥5 for at least 80 % of the elements of the partition. We impose a more conservative criterion and require E_XY-NULL_(i, j) ≥5 in all elements. N_E_ is the largest positive integer satisfying this criterion. We have previously derived an expression for E_XY-NULL_(i, j) for an equi-probable partition of the X and Y axes. Our criterion on E_XY-NULL_(i, j) becomes

$$ {\text{E}}_{{{\text{XY}} - {\text{NULL}}}} ({\text{i}},{\text{j}}) = {\text{N}}_{\text{D}} /{\text{N}}_{\text{E}}^{2} \ge 5 $$

N_E_ is the largest integer meeting the criterion $({\text{N}}_{\text{D}} /5)^{1/2} \ge {\text{N}}_{\text{E}}$. If, for example, N_D_ = 8,192, then $({\text{N}}_{\text{D}} /5)^{1/2} = 40.447$ and N_E_ = 40. O_X_(i) and O_Y_(j) will be either 204 or 205. The between bin differences of 204 or 205 occur because 8,192 is not a multiple of 40. The upper and lower bound of each element of the partition are varied to give the best possible approximation of P_X_(i) = P_Y_(j) = 1/N_E_. When the bin assignments of X and Y values in the time series are known, P_XY_(i, j) can be determined. The estimate of mutual information and the probability of the null hypothesis then follow from the previous formulas.

## References

[CR1] Albano AM, Bedonie C, Cellucci CJ, Halkides D, Miller V, Ree J, Turruella A, Harner R, Rapp PE, Sreenivasan N, Pradhan RN, Rapp PE (1999). Spatiotemporal EEG information transfer in an episode of epilepsy. Nonlinear dynamics and brain functioning.

[CR2] Albano AM, Cellucci CJ, Harner RJ, Rapp PE, Mees AI (2000). Optimization of embedding parameters for prediction of seizure onset with mutual information. Nonlinear dynamics and statistics.

[CR3] Barlow JS (1997). The early history of EEG data-processing at the Massachusetts Institute of Technology and the Massachusetts General Hospital. Int J Psychophysiol.

[CR4] Bartolomei F, Bosma I, Klein M, Baayen JC, Reijneveld JC, Postma TJ, Heimans JJ, van Dijk BW, deMunck JC, Jongh A, Cover KS, Stam CJ (2006). Distributed functional connectivity in brain tumour patients: evaluation by graph analysis and synchronization matrices. Clin Neurophysiol.

[CR5] Belmonte MK, Allen G, Beckel-Mitchner A, Boulanger LM, Carper RA, Webb SH (2004). Autism and abnormal development of brain connectivity. J Neurosci.

[CR6] Boashash B (1992). Estimating and interpreting the instantaneous frequency of a signal. Proc IEEE.

[CR7] Bosma I, Douw L, Bartolomei F, Heimans JJ, van Dijk BW, Postma J, Stam CJ (2008). Synchronized brain activity and neurocognitive function in patients with low grade glioma: a magnetoencephalography study. Neuro-Oncology.

[CR8] Brazier MAB, Casby JU (1952). Cross correlation and autocorrelation studies of electroencephalographic potentials. Electroencephalogr Clin Neurophysiol.

[CR9] Breakspear M, Terry JR, Friston KJ, Harris AWF, Williams LM, Brown K, Brennan J, Gordon E (2003). A disturbance of nonlinear interdependence in scalp EEG of subjects with first episode schizophrenia. Neuroimage.

[CR10] Breitung J, Swanson NR (2002). Temporal aggregation and spurious instantaneous causality in multiple time series models. J Time Ser Anal.

[CR11] Broca P (1861) Remarks on the seat of the faculty of articulated language following an observation of aphemia (loss of speech). Bulletin de la Société Anatomique 6:330–357 (translation C. D. Green, Classics in the History of Psychology, 2000)

[CR12] Cabestang F, Vaughan TM, McFarland DJ, Wolpaw JR (2007). Classification of evoked potentials by Pearson’s correlation in a brain-computer interface. AMSE Model Ser C Autom Control Theory Appl.

[CR13] Callaway E, Harris PR (1974). Coupling between cortical potentials from different areas. Science.

[CR14] Cao C, Slobounov S (2010). Alteration of cortical functional connectivity as a result of traumatic brain injury revealed by graph theory, ICA and sLORETA analyses of EEG signals. IEEE Trans Neural Syst Rehabil Eng.

[CR15] Castellanos NP, Paúl N, Ordóñez VE, Demuynck O, Bajo R, Campo P, Bilbao A, Ortiz T, del-Pozo F, Maetsú F (2010). Reorganization of functional connectivity as a correlate of cognitive recovery in acquired brain injury. Brain.

[CR16] Castellanos NP, Leyva I, Buldú JM, Bajo R, Paúl N, Cuesta P, Ordóñez VE, Pascua CL, Bocaletti S, Maestú E, del-Pozo F (2011). Principles of recovery from traumatic brain injury: reorganization of functional networks. Neuroimage.

[CR17] Castellanos NP, Bajo R, Cuesta P, Villacorta-Atienza JA, Paúl N, Garcia-Prieto J, Del-Pozo F, Maestú F (2011). Alteration and reorganization of functional networks: a new perspective in brain injury study. Front Hum Neurosci.

[CR18] Cellucci CJ, Albano AM, Rapp PE (2005) Statistical validation of mutual information calculations: comparisons of alternative numerical algorithms. Phys Rev E 71:066208-1–066208-14 10.1103/PhysRevE.71.066208 16089850

[CR19] Chen F, Xu J, Gu F, Yu X, Meng X, Qiu Z (2000). Dynamic processes of information transmission complexity in human brains. Biol Cybern.

[CR20] Cochran WG (1954). Some methods for strengthening the common χ^2^ test. Biometrics.

[CR21] Cover TM, Thomas JA (1991). Elements of information theory.

[CR22] Dauwels J, Vialatte F, Cichocki A (2010). A comparative study of synchrony measures for the early detection of Alzheimer’s disease based on EEG. Neuroimage.

[CR23] Douw L, Dellen E, Baayen JC, Klein M, Velis DN, Alpherts WCJ, Heimans JJ, Reijneveld JC, Stam CJ (2010) The lesioned brain: still a small world? Front Hum Neurosci 4, Article 174:1–9 10.3389/fnhum.2010.00174 PMC2991225 21120140

[CR24] Duckrow RB, Albano AM (2003) Comment on “Performance of different synchronization measures in real data: a case study on electroencephalographic signals”. Phys Rev E 67 063901-1–063901-3 10.1103/PhysRevE.67.063901 16241284

[CR25] Filligoi G, Padalino M, Pioli S (2011) A Matlab software for detection and counting of epileptic seizures in 72 hours Holter-EEG. Cyber J Sel Areas Bioeng 1:1–8

[CR26] Fingelkurts AA, Fingelkurts AA, Kähkönen S (2005). Functional connectivity in the brain—is it an elusive concept?. Neurosci Biobehav Rev.

[CR27] Fraser AM, Swinney HL (1986). Independent coordinates for strange attractors from mutual information. Phys Rev A.

[CR28] Friston K, Frith CD, Frackowiak RSJ (1993). Time-dependent changes in effective connectivity measured with PET. Hum Brain Mapp.

[CR29] Friston KJ, Frith CS, Liddle PF, Frackowiak RSJ (1993). Functional connectivity: the principal component analysis of large (PET) data sets. J Cereb Blood Flow Metab.

[CR30] Georgopoulos AP, Karageorgiou E, Leuthold AC, Lewis SM (2007). Synchronous neural interactions assessed by magnetoencephalography: a functional biomarker for brain disorders. J Neural Eng.

[CR31] Geschwind N, Devinsky O, Schachter S (1997). The paradoxical position of Kurt Goldstein in the history of aphasia. Norman Geschwind: selected publications on language, epilepsy and behavior.

[CR32] Gevins AS, Gevins AS, Rémond A (1987). Correlation analysis. Methods of analysis of brain electrical and magnetic signals. EEG handbook (revised series vol 1).

[CR33] Goldstein K (1927). Die Lokalisation in der Großhirnrinde nach den Erfahrungen am kranken Menschen.

[CR34] Goldstein K (1934, 2000) Der Aufbau des Organismus. Einführung in die Biologie unter besonderer Berücksichtigung der Erfahrungen am kranken Menschen. Nijhof, The Hague (Republished in English as The Organism, Forward by Oliver Sachs. Zone Books, Brooklyn, NY)

[CR35] Granger CWJ (1969). Investigating causal relations by econometric models and cross-spectral methods. Econometrica.

[CR36] Grefkes C, Fink GR (2012). Disruption of motor network connectivity post-stroke and its noninvasive neuromodulation. Curr Opin Neurol.

[CR37] Guevara R, Pérez Velazquez JL, Nenadovic V, Wennberg R, Senjanović G, Dominguez LG (2005). Phase synchronization measurements using electroencephalographic recordings. What can we really say about neuronal synchrony?. Neuroinformatics.

[CR38] Güntekin B, Saatci E, Yener G (2008). Decrease of evoked delta, theta and alpha coherences in Alzheimer patients during a visual oddball task. Brain Res.

[CR39] Ham TE, Sharp DJ (2012). How can investigation of network function inform rehabilitation after traumatic brain injury?. Curr Opin Neurol.

[CR40] He Z, Maekawa K (2001). On spurious Granger causality. Econ Lett.

[CR41] Herrmann CS, Demiralp T (2005). Human EEG gamma oscillations in neuropsychiatric disorders. Clin Neurophysiol.

[CR42] Horowitz B (2003). The elusive concept of brain connectivity. Neuroimage.

[CR43] Hughlings-Jackson J (1874, 1958) On the nature of the duality of the brain. In: Taylor J (ed) Selected writings of John Hughlings-Jackson, vol 2. Basic Books, New York, pp 129–146

[CR44] Hughlings-Jackson J (1882, 1958) On some implications of dissolution of the nervous system. In: Taylor J (ed) Selected writings of John Hughlings-Jackson, vol 2. Basic Books, New York, pp 29–45

[CR45] Hurtado JM, Rubchinskiy LL, Sigvardt KA (2004). Statistical method for detecting of phase locking episodes in neural oscillations. J Neurophysiol.

[CR46] Hyvärinen A, Karhunen J, Oja E (2001). Independent component analysis.

[CR47] Imahori K, Suhara K (1949). On the statistical method in brain wave study. Folia Psychiatrica et Neurological Japonica.

[CR48] Inouye T, Shinosaki K, Yagasaki A (1983). The direction of spread of alpha activity over the scalp. Electroencephalogr Clin Neurophysiol.

[CR49] Inouye T, Shinosaki K, Iyama A, Matsumoto Y (1993). Localization of activated areas and directional EEG patterns during mental arithmetic. Electroencephalogr Clin Neurophysiol.

[CR50] Just MA, Cherkassky VL, Keller TA, Minshew JW (2004). Cortical activation and synchronization during sentence comprehension in high-functioning autism: evidence of underconnectivity. Brain.

[CR51] Kana RK, Keller TA, Minshew N, Just MA (2007). Inhibitory control in high function autism: decreased activation and underconnectivity in inhibition networks. Biol Psychiatry.

[CR52] Kaneko K (1986). Lyapunov analysis and information flow in coupled map lattices. Physica D.

[CR53] Kasahara M, Menon DK, Salmond CH, Outtrim JG, Taylor Tavares JV, Carpenter TA, Pickard JD, Sahakian BJ, Stamatakis EA (2010). Altered functional connectivity in the motor network after traumatic brain injury. Neurology.

[CR54] Khan S, Bandyopadhyay S, Ganguly AR, Saigal S, Erickson DJ, Protopopescu V, Ostrouchov G (2007). Relative performance of mutual information estimation methods for quantifying the dependence among short noisy data. Phys Rev E.

[CR55] Knuth DE (1981). Seminumerical algorithms. Volume 2 of the art of computer programming.

[CR56] Kumar R, Gupta RK, Husain M, Chaudhry C, Srivastava A, Saksensa S, Rathore RK (2009). Comparative evaluation of corpus callosum DTI metrics in acute mild and moderate traumatic brain injury: its correlation with neuropsychometric tests. Brain Inj.

[CR57] Kutas M, McCarthy G, Donchin E (1977). Augmenting mental chronometry: the P300 as a measure of stimulus evaluation time. Science.

[CR58] Lanius RA, Wiliamson PC, Densmore M, Boksman K, Neufeld RW, Gati JS, Menon RS (2004). The nature of traumatic memories: a 4-T fMRI functional connectivity analysis. Am J Psychiatry.

[CR59] Latora V, Marchiori M (2001). Efficient behavior of small world networks. Phys Rev Lett.

[CR60] Lawrie SM, Buechel C, Whalley HC, Frith CD, Friston KJ, Johnstone EC (2002). Reduced frontotemporal functional connectivity in schizophrenia associated with auditory hallucinations. Biol Psychiatry.

[CR61] Lee L, Harrison LM, Mechelli A (2003). A report of the functional connectivity workshop Dusseldorf 2002. Neuroimage.

[CR62] Lenne B, Blanc JL, Nandrino JL, Gallois P, Hautecoeur P, Pezard L (2012) Decrease of mutual information in brain electrical activity of patients with relapsing-remitting multiple sclerosis. Behav Neurol. 12 April 2012 [Epub ahead of print] 10.3233/BEN-120278 PMC5214621 23242355

[CR63] Locatelli T, Cursi M, Liberati D, Franceschi J, Comi G (1998). EEG coherence in Alzheimers disease. Electroencephalogr Clin Neurophysiol.

[CR64] Lopes da Silva FH, Niedermeyer E, Lopes da Silva FH (1987). EEG analysis: theory and practice. Electroencephalography basic principles, clinical applications and related fields.

[CR65] Ludwig D (2012). Language and human nature: Kurt Goldstein’s neurolinguistic foundation of a holistic philosophy. J Hist Behav Sci.

[CR66] Lynall M-E, Bassett DS, Kerwin R, McKenna PJ, Kitzbichler M, Muller U, Bullmore E (2010). Functional connectivity and brain networks in schizophrenia. J Neurosci.

[CR67] Madulara MD, Francisco PAD, Nawang S, Arogancia DC, Cellucci CJ, Rapp PE, Albano AM (2012). EEG transfer entropy tracks changes of information transfer on the onset of vision. Int J Mod Phys.

[CR68] Mars NJI, Lopes da Silva FH (1983). Propagation of seizure activity in kindled dogs. Electroencephalogr Clin Neurophysiol.

[CR69] Mars NJI, Lopes da Silva FH, Gevins AS, Rémond A (1987). EEG analysis methods based on information theory. Methods of analysis of brain electrical and magnetic signals. EEG handbook (revised series, vol 1).

[CR70] Mars NJI, Thompson PM, Wilkus RJ (1985). Spread of epileptic seizure activity in humans. Epilepsia.

[CR71] McGillem CD, Aunou JI, Gevins AS, Rémond A (1987). Analysis of event-related potentials. Methods of analysis of brain electrical and magnetic signals. EEG handbook (revised series vol 1).

[CR72] Michelyannis S, Pachou E, Stam CJ, Breakspear M, Bitsios P, Vourkas M, Erimaki S, Zervakis M (2006). Small-world networks and disturbed functional connectivity in schizophrenia. Schizophr Res.

[CR73] Mullen KM, Vohr BR, Katz KH, Schneider KC, Lacadie C, Hampson M, Makuch RW, Reiss AL, Constable RT, Ment LR (2011). Preterm birth results in alterations in neural connectivity at age 16 years. Neuroimage.

[CR74] Murias M, Webb SJ, Greenson J, Dawson G (2007). Resting state cortical connectivity reflected in EEG coherence in individuals with autism. Biol Psychiatry.

[CR75] Nakamura T, Hillary FG, Biswal BB (2009). Resting network plasticity following brain injury. PLoS One.

[CR76] Nolte G, Bai O, Wheaton L, Mari Z, Vorbach S, Hallet M (2004). Identifying true brain interaction from EEG data using the imaginary part of coherency. Clin Neurophysiol.

[CR77] Nunez PL, Srinivasan R, Westdorp AF, Wijesinghe RS, Tucker DM, Silberstein RB, Cadusch PJ (1997). EEG coherency. I. Statistics, reference electrode, volume conduction, Laplacians, cortical imaging and interpretation at multiple scales. Electroencephalogr Clin Neurophysiol.

[CR78] Nunez PL, Silberstein RB, Shi ZP, Carpenter MR, Srinivasan R, Tucker DM, Doran SM, Cadusch PJ, Wijesinghe RS (1999). EEG coherency: II. Experimental comparisons of multiple measures. Clin Neurophsyiol.

[CR79] Park SK, Miller KW (1988). Random number generators. A good one is hard to find. Commun ACM.

[CR80] Pascual-Marqui RD (2002). Standardized low-resolution brain electromagnetic tomography (sLORETA): technical details. Methods Find Exp Clin Pharmacol.

[CR81] Pascual-Marqui RD, Esslen M, Kochi K, Lehmann D (2002). Functional imaging with low resolution brain electromagnetic tomography (LORETA): a review. Methods Find Exp Clin Pharmacol.

[CR82] Pikovsky A, Rosenblum M, Kurths J (2001). Synchronization: a universal concept in nonlinear sciences.

[CR83] Ponten SC, Bartolemi F, Stam CJ (2007). Small-world networks and epilepsy: graph theoretical analysis of intracerebrally recorded mesial temporal lobe seizures. Clin Neurophysiol.

[CR84] Press WH, Flannery BP, Teukolsky SA, Vetterling WT (1992). Numerical recipes. The art of scientific computing.

[CR85] Quian Quiroga R, Kraskov A, Kreuz T, Grassberger P (2002) Performance of different synchronization measures in real data: a case study on electroencephalographic signals. Phys Rev E 65:041903-1–041903-13 10.1103/PhysRevE.65.041903 12005869

[CR86] Reshef DN, Reshef YA, Finucane HK, Grossman SR, McVean G, Turnbaugh PJ, Lander ES, Mitzenmacher M, Sabeti PC (2011). Detecting novel associations in large data sets. Science.

[CR87] Rippon G, Brock J, Brown C, Boucher J (2006). Disordered connectivity in the autistic brain: challenges for the ‘new psychophysiology’. Int J Psychophysiol.

[CR88] Rosenbaum RS, Furey ML, Horwitz B, Grady CL (2008). Altered connectivity among emotion-related brain regions during short-term memory in Alzheimer’s disease. Neurobiol Aging.

[CR89] Rubinov M, Knock SA, Stam CJ, Micheloyannis S, Harris AWF, Williams LM, Breakspear M (2009). Small world properties of nonlinear brain activity in schizophrenia. Hum Brain Mapp.

[CR90] Sazonov AV, Ho CK, Bergmans JWM, Arends JBAM, Griep PAM, Verbitskiy EA, Cluitmans PJM, Boon PAJM (2009). An investigation of phase locking index for measuring interdependency of cortical source signals recorded in the EEG. Biol Cybern.

[CR91] Schiff SJ (2005). Dangerous phase. Neuroinformatics.

[CR92] Schnitzler A, Gross J (2005). Normal and pathological oscillatory communication in the brain. Nat Rev Neurosci.

[CR93] Schreiber T (2000). Measuring information transfer. Phys Rev Lett.

[CR94] Shaw M (2002). Abnormal functional connectivity in posttraumatic stress disorder. Neuroimage.

[CR95] Sims C (1972). Money, income and causality. Am Econ Rev.

[CR96] Smith-Bassett D, Bullmore E (2006). Small-world brain networks. Neuroscientist.

[CR97] Spencer KM, Handy TC (2005). Averaging, detection and classification of single-trial ERPs. Event related potentials. A methods handbook.

[CR98] Sponheim SR, McGuire KA, Kang SS, Davenport ND, Aviyente S, Bernat EM, Lim KO (2011). Evidence of disrupted functional connectivity after combat related blast injury. Neuroimage.

[CR99] Sporns O, Honey CJ (2006). Small worlds inside big brains. Proc Natl Acad Sci USA.

[CR100] Stam CJ (2005). Nonlinear dynamical analysis of EEG and MEG: review of an emerging field. Clin Neurophysiol.

[CR101] Stam CJ, Reijneveld JC (2007) Graph theoretical analysis of complex networks in the brain. BMC Nonlinear Biomed Phys 1(3). doi:10.1186/1753-4631-1-3 10.1186/1753-4631-1-3 PMC1976403 17908336

[CR102] Stam CJ, van Djik BW (2002). Synchronization likelihood: an unbiased measure of generalized synchronization in multivariate data sets. Physica D.

[CR103] Stam CJ, Jones BF, Manshanden I, van Cappellen van Walsum AM, Montez T, Verbunt JP, de Munck JC, van Dijk BW, Berendse HW, Scheltens P (2006). Magnetoencephalographic examination of resting-state functional connectivity in Alzheimer’s disease. Neuroimage.

[CR104] Stam CJ, Jones BF, Nolte G, Breakspear M, Scheltens PH (2007). Small-world networks and functional connectivity in Alzheimer’s disease. Cereb Cortex.

[CR105] Stam CJ, Nolte G, Daffertshofer A (2007). Phase lag index: assessment of functional connectivity from multichannel EEG and MEG with diminished bias from common sources. Hum Brain Mapp.

[CR106] Stam CJ, de Haan W, Daffertshofer A, Jones BF, Manshanden I, van Cappellen van Walsum AM, Montez T, Verbunt JPA, de Munck JC, van Dijk BW, Berendse HW, Scheltens P (2009). Graph theoretical analysis of magentoencephalographic functional connectivity in Alzheimer’s disease. Brain.

[CR107] Symond MB, Harris AWF, Gordon E, Williams LM (2005). “Gamma synchrony” in first-episode schizophrenia: a disorder of temporal connectivity?. Am J Psychiatry.

[CR108] Takens F, Rand DA, Young LS (1981). Detecting strange attractors in turbulence. Lecture notes in mathematics.

[CR109] Tesak J, Code C (2008). Milestones in the history of aphasia: theories and protagonists.

[CR110] Triola MF (2008). Elementary statistics.

[CR111] Tsirka V, Simos PG, Vakis A, Kanatsouli K, Vourkas M, Erimaki S, Pauchou E, Stam CJ, Micheloyannis S (2011). Mild traumatic brain injury: graph-model characterization of brain networks for episodic memory. Int J Psychophysiol.

[CR112] Uhlhaas PJ, Singer W (2006). Neural synchrony in brain disorders: relevance for cognitive dysfunctions and pathophysiology. Neuron.

[CR113] van Dellen E, Douw L, Baayen JC, Heimans JJ, Ponten SC, Vandertop WB, Velis DN, Stam CJ,. Reijneveld JC (2009) Long-term effects of temporal lobe epilepsy on local neural networks: a graph theoretical analysis of corticography recordings. PLoS One 4(11):e8081-1–e8081-9 10.1371/journal.pone.0008081 PMC2778557 19956634

[CR114] Vastano JA, Swinney HL (1988). Information transport in spatiotemporal systems. Phys Rev Lett.

[CR115] Vidal C, Nicolson R, DeVito TJ, Hayashi KM, Geaga JA, Drost DJ, Williamson PC, Rajakumar N, Sui Y, Dutton RA, Toga AW, Thompson PM (2006). Mapping corpus callosum deficits in autism: an index of aberrant cortical connectivity. Biol Psychiatry.

[CR116] Watts DJ, Strogatz SH (1998). Collective dynamics of small-world networks. Nature.

[CR117] Wendling F, Ansari-Asl K, Bartolomei F, Senhadji L (2009). From EEG signals to brain connectivity: a model based evaluation of interdependence measures. J Neurosci Methods.

[CR118] Wernicke C, Church A (1908). The symptom-complex of aphasia. Diseases of the nervous system.

[CR119] Wiener N, Beckenbach EF (1956). The theory of prediction. Modern mathematics for engineers.

[CR120] Woody CD (1967). Characterization of an adaptive filter for the analysis of variable latency neuroelectric signals. Med Biol Eng.

[CR121] Xu J, Liu Z-R, Liu R, Yang Q-F (1997). The information transmission of human brain cortex. Physica D.

[CR122] Yagi A, Bali L, Callaway E (1976). Optimum parameters for measurement of cortical coupling. Physiol Psychol.

[CR123] Zeitlinger S (2001). Kurt Goldstein: a philosophical scientist. J Hist Neurosci.

[CR124] Zhou YX, Dougherty JH, Hubner KF, Bai B, Cannon RL, Hutson RK (2008). Abnormal connectivity in the posterior cingulate and hippocampus in early Alzheimer’s disease and mild cognitive impairment. Alzheimers Dement.

[CR125] Zouridakis G, Patidar U, Pollonini L, Situ N, Rezaie R, Castillo EM, Levin HS, Papanicolaou AC (2011) Default brain connectivity network in mild traumatic brain injury—preliminary MEG results. (MECBME) 2011 1st middle east conference on biomedical engineering

